# Precise identification of cancer cells from allelic imbalances in single cell transcriptomes

**DOI:** 10.1038/s42003-022-03808-9

**Published:** 2022-09-07

**Authors:** Mi K. Trinh, Clarissa N. Pacyna, Gerda Kildisiute, Christine Thevanesan, Alice Piapi, Kirsty Ambridge, Nathaniel D. Anderson, Eleonora Khabirova, Elena Prigmore, Karin Straathof, Sam Behjati, Matthew D. Young

**Affiliations:** 1 Wellcome Sanger Institute, Wellcome Genome Campus, Hinxton, Cambridge UK; 2 University College London Great Ormond Street Institute of Child Health, London, UK; 3 Cambridge University Hospitals NHS Foundation Trust, Cambridge, UK; 4 Department of Paediatrics, University of Cambridge, Cambridge, UK

**Keywords:** Data processing, Cancer genomics, Genomics, Sequencing

## Abstract

A fundamental step of tumour single cell mRNA analysis is separating cancer and non-cancer cells. We show that the common approach to separation, using shifts in average expression, can lead to erroneous biological conclusions. By contrast, allelic imbalances representing copy number changes directly detect the cancer genotype and accurately separate cancer from non-cancer cells. Our findings provide a definitive approach to identifying cancer cells from single cell mRNA sequencing data.

## Abstract (web summary)

The identification of cancer cells from single cell transcriptomes can be improved by detecting allelic imbalances due to copy number changes.

## Introduction

Single cell mRNA sequencing has enabled transcriptomic profiling of tumours and their environment with data being generated across the entire spectrum of human cancer. Studying the cancer transcriptomes depends on accurate identification of cancer cells. Therefore, the foundational step of tumour single cell analyses is separating cancer from non-cancer cells.

The simplest approach to identifying cancer cells is to use expression of cancer specific marker genes. However, such genes do not always exist and are generally insufficiently precise, especially without corroborating readouts such as cellular morphology. Another approach is to infer the presence of tumour-defining somatic copy-number changes from shifts in average expression^1,2^. The idea here is that gains or losses of genomic regions will generally increase or decrease the expression level of genes in these regions respectively. Challenges with this approach include smoothing and denoising expression changes, establishing a baseline against which to measure shifts in expression, segmenting the genome, and identifying changes in expression not due to copy-number changes. Despite these challenges, both marker genes and shifts in average expression, which we collectively refer to as “expression-based annotation”, may accurately identify cancer cells in certain circumstances. However, if there is any novelty or ambiguity in the identity of cancer cells, then these two approaches are inherently fallible as they are both based on expression and not direct evidence that a cell is cancerous, i.e. that it carries the somatic cancer genome.

For example, there has been historical controversy about what cell types are malignant in neuroblastoma, a childhood cancer that arises from peripheral nervous sympathetic lineages. In addition to unambiguous cancer cells, neuroblastomas often harbour stromal cells, composed of Schwannian stroma or mesenchymal cells. It has been suggested that these stromal cell types represent cancer lineages, although a rich body of evidence, including cytogenetic investigations, have not supported this proposition^3^. Recent single cell mRNA studies of neuroblastoma have rekindled the debate on the basis of expression-based cancer cell identification^4^. Although neuroblastoma is an exemplar of the difficulties in annotating single cell tumour transcriptomes, the same problems are common to tumours with complex histology or unresolved origins. Even among tumours with well-defined origins, the variability inherent to all cancer can make annotation challenging.

**Fig. 1:**
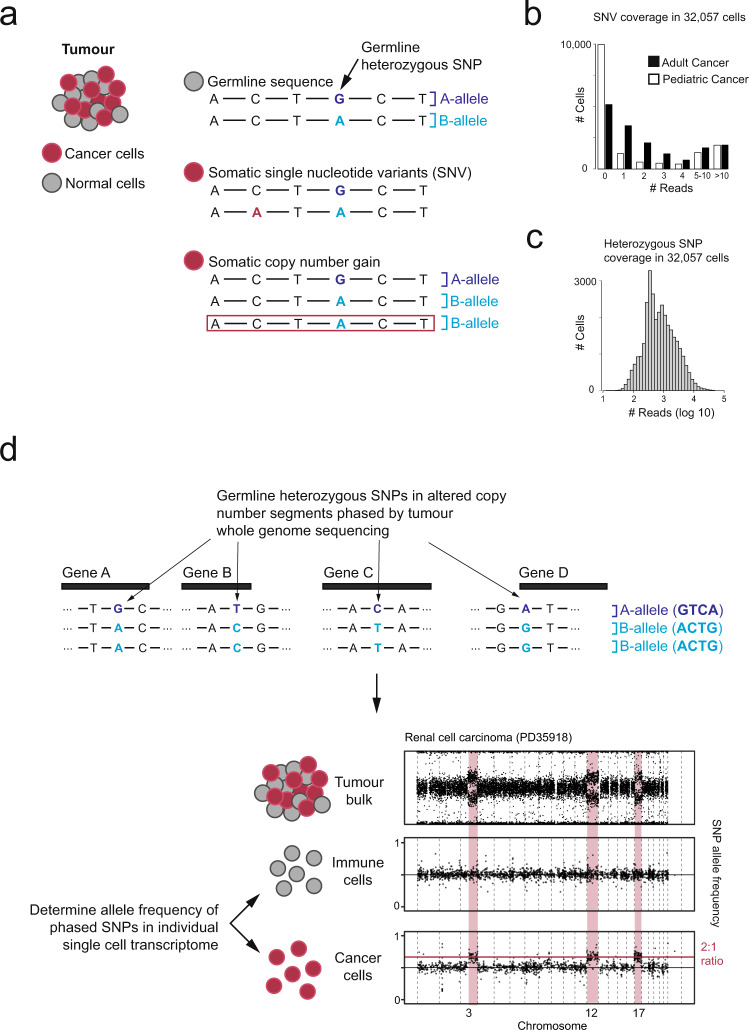
Overview of different approaches to identifying cancer-derived cells. **a** Genomic changes present in cancer genomes. **b** Number of cells (y-axis) with N reads covering point mutations (x-axis), separated by low (NB neuroblastoma) and high (RCC renal cell carcinoma) mutation burden. **c** Number of cells (y-axis) with N reads covering heterozygous single nucleotide polymorphisms (SNP) (x-axis). **d** Overview of using allelic shifts representing copy-number changes to detect cancer cells.

The alternative to expression-based annotation is direct detection of either cancer-defining (i.e. somatic) point mutations or copy-number aberrations from the nucleic acid sequences of each transcriptome, which we pursued here. Such approaches utilise additional information from whole genome/exome sequencing of tumour DNA to detect the altered genotype or the allelic imbalance it creates. More specifically, sequencing of tumour DNA is used to identify regions of copy-number change shared by all cancer cells. Within these regions the B-allele frequency or BAF, defined as the fraction of reads from the non-reference allele, will differ from the value of 0.5 that characterises normal cells (Fig. 1a). The altered BAF is then used to phase together heterozygous bases across the altered region and the nucleotide sequences underlying single transcriptomes can be interrogated for these cancer-defining shifts. The principle of using shifts in BAF to detect copy-number changes has been previously used to detect de novo copy-number changes in single cell data^5,6^. Here we leverage the extra information provided by tumour DNA sequencing to use shifts in BAF to precisely identify single cancer cell transcriptomes.

## Results

Briefly, our method, which we call alleleIntegrator, works as follows. Firstly, whole genome or exome sequencing is performed on tumour DNA. From this, regions of copy-number change are identified, using established methods such as ASCAT^7^, along with germline heterozygous single nucleotide polymorphisms (SNPs) within altered regions. The alleles with frequency significantly greater than 0.5 (binomial test) are phased together and collectively designated the “major allele”. The allele frequency of all phased heterozygous SNPs within copy-number altered regions is then measured in each single cell transcriptome. Finally, the posterior probability of both the normal genotype (where all alleles have BAF 0.5) and the cancer genotype (where the BAF of each allele matches that implied by the copy-number status of the cancer) are calculated. It is possible that allelic shifts may result from allele-specific expression rather than copy-number change. To control for this, we exclude genes known to be imprinted or have allele-biased expression (e.g. *HLA* genes), model any residual allele-specific expression using the data, and only consider large regions spanning multiple genes. Those cells with a posterior probability exceeding some threshold (set to 99% throughout this paper) are designated as cancer or normal cells, with all other cells designated as unassigned.

To test approaches used to identify cancer cells, we generated or downloaded single cell droplet-based 3′ single cell transcriptomes from 13 individuals and 5 tumour types: renal cell carcinoma (RCC), neuroblastoma, Wilms tumour, Ewing’s sarcoma, and atypical teratoid rhabdoid tumour (AT/RT)^8–10^ (Supplementary Table 1). We first tested if detection of cancer specific point mutations would identify cancer transcriptomes. Across all samples, the majority of cells had no reads covering a point mutation (Fig. 1b), with on average 9.7 reads per ten thousand point mutations per cell (range 0 to 556). This implies that identifying cancer cells from point mutations is possible, but depends on the mutation burden being high and the cost of false negatives being low. By contrast, an average of 1522 reads per cell covered heterozygous single nucleotide polymorphisms (SNPs), implying 0.5 informative reads per megabase per transcriptome (Fig. 1c). As copy-number changes may alter the allelic ratio, these data can be used to detect the cancer genotype (Fig. 1d). This implies that a loss of heterozygosity (LoH) of 19.7 megabases or more should be detectable in single transcriptomes (assuming a binomial distribution and 99% accuracy).

Next, we compared the performance of cancer transcriptome identification using expression- and nucleotide-based copy-number detection. For each patient we ran three copy-number detection methods, CopyKAT^2^, inferCNV^1^, and a statistical model based on allelic ratios^8^, which we named alleleIntegrator. We evaluated how well each method recovered the true copy-number profile and cancer cell transcriptomes. As inferCNV does not call cancer cell transcriptomes, we evaluated this method on its copy-number profile only.

**Fig. 2:**
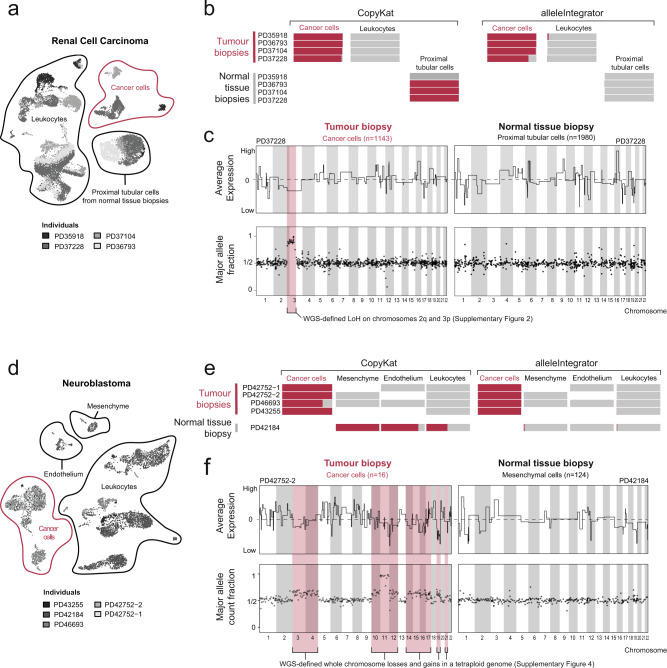
Comparison of cancer cell annotation and copy-number profile using allelic-ratio and expression-based approaches. **a** UMAP of RCC single cell transcriptomes showing patient (shading), cell type (contours and labels), and patient composition (barplots). Inset shows expression of RCC marker *CA9*. PTC proximal tubular cells derived from normal biopsies. **b** Cancerous (red) and non-cancerous (grey) cell fraction excluding ambiguous cells by cell type (x-axis) and sample/region (y-axis) called by CopyKAT (left) or alleleIntegrator (right). **c** Copy-number profile for PD37228 tumour (left) and proximal tubular (right) clusters from normalised averaged expression (top panels, solid black line) and allelic ratio (bottom panel, one dot per bin with ~500 reads), with true copy-number changes from DNA (red shading). **d**–**f** As per (**a**–**c**) but for neuroblastoma.

We first considered RCC, an adult kidney cancer where the cancer cell transcriptome can be definitively identified based on the tumour marker *CA9*, caused by the near universal disruption of the *VHL* gene underpinning RCC^11^ (Fig. 2a). For each individual, we used single cell transcriptomes from both tumour biopsies expressing *CA9* and from macroscopically and histologically normal tissue biopsies from uninvolved regions of the kidney that did not express *CA9*. This guaranteed that the assumptions of inferCNV and CopyKAT, that a mixture of cancer and normal transcriptomes be present, were satisfied. Despite this, CopyKAT’s expression-based identification classed 98% (2953) proximal tubular cells derived from normal tissue as cancerous, compared to 0.2% (4 cells) identified as cancer-derived by allelic ratio (Fig. 2b, Supplementary Fig. 1). As proximal tubular cells are the probable cell of origin for RCC, it is likely that expression-based copy-number inference incorrectly identified proximal tubular cells as cancer-derived due to their transcriptional similarity to RCC cells. Amongst the 1718 verified cancer cells, expression-based identification called 1096 as tumour and 35 as normal, while alleleIntegrator identified 712 as tumour and 41 as normal (with the remaining cells unassigned).

We next assessed how well the inferred copy-number profiles matched the ground truth—i.e. somatic copy-number profiles obtained from whole genome sequences—at the chromosome level. There is a good visual agreement between the ground truth profile and allelic ratios, while both CopyKAT and inferCNV exhibit deviations from the expected values (Fig. 2c, Supplementary Fig. 2). To quantify this comparison, we designate regions as changed or neutral based on an expression cut-off, which we compared to the ground truth. Varying this cut-off produced a receiver operating characteristic (ROC) curve for each method, with average area 0.97 for alleleIntegrator, 0.87 for CopyKAT, and 0.74 for inferCNV (Supplementary Fig. 3). In aggregate, these analyses demonstrate the potential for expression-based methods to misidentify normal cells as cancerous, illustrating their shortcomings in identifying novel cancer cell types.

As a contrast to RCC, we tested cancer transcriptome identification on single cell transcriptomes from neuroblastomas, which have no definitive single marker equivalent to *CA9* in RCC (Fig. 2d). As before, both expression- and allelic ratio-based identification identified tumour cells accurately (Fig. 2e, Supplementary Fig. 1). As neuroblastoma lacks a definitive marker gene, it cannot be known if cancer cell transcriptomes have been captured before expression-based copy-number inference is run. To consider what would happen if an experiment did not capture cancer cells, we ran all methods on sample PD42184, which is derived from a normal adrenal gland and therefore contains no tumour cells. Expression-based copy-number inference predicted 1926 cancer-derived cells, including mesenchymal cells (Fig. 2e). By contrast, these cells are identified as normal based on their allelic ratio (Fig. 2e). The expression-based copy-number profiles are also consistent with the mesenchymal cells being cancer derived, with shifts in average expression on chromosomes 1,2,3 and 12 (Fig. 2f, Supplementary Fig. 4). As with RCC, this was part of a larger pattern where expression-based profiles only weakly matched the ground truth, while allelic ratios captured the truth with high accuracy despite the complex copy-number profiles, yielding average ROC areas of 0.89 for alleleIntegrator, 0.28 for CopyKAT, and 0.28 for inferCNV (Supplementary Fig. 3). Overall, this demonstrates the risk of drawing erroneous biological conclusions, in this case that mesenchymal cells are cancer-derived, when relying on expression-based copy-number inference of cancer transcriptomes.

**Fig. 3:**
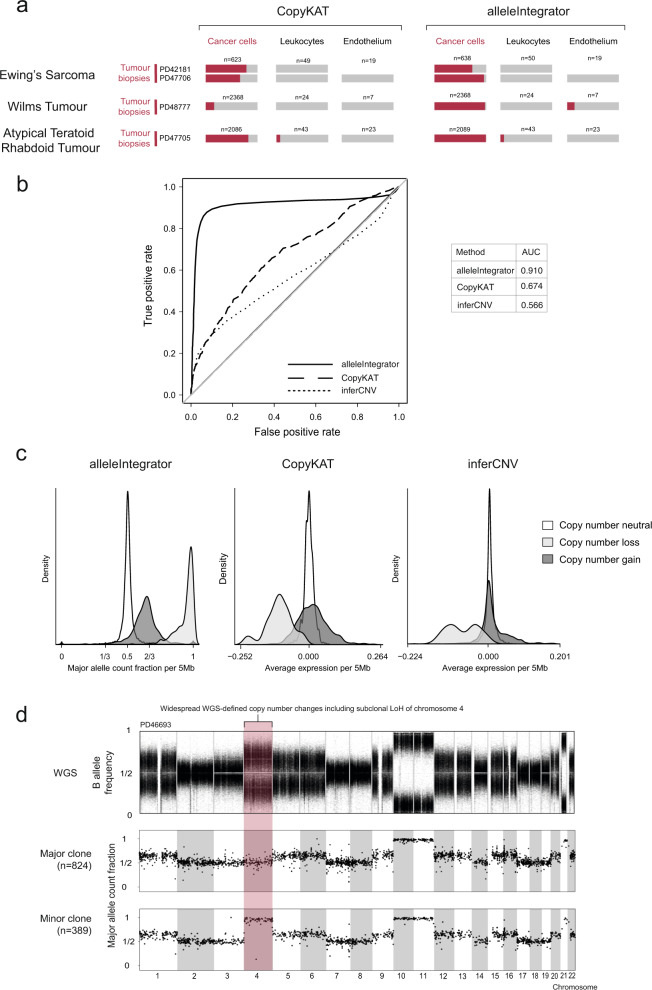
alleleIntegrator accurately recovers copy-number profile and clonal structure for a wide range of tumour types. **a** Fraction of cells called cancerous (red) and non-cancerous (grey), excluding ambiguous cells, by cell type (x-axis) and tumour type (y-axis), called by alleleIntegrator (left) or CopyKAT (right). **b** Receiver operating characteristic (ROC) curve for all individuals measuring the sensitivity and specificity with which different methods (line type) recover the true copy-number profile. The table on the right shows the total area under the curve for each method. **c** Distribution across all individuals and regions of allelic ratios (left) or averaged expression values (middle and right) in 5 megabase regions that contain copy-number gains (dark shading), loss (intermediate shading), or are copy-number neutral (white). **d** Allelic ratios (y-axis) across the genome (x-axis) from bulk tumour DNA (top panel), cells assigned to the major clone (middle panel) and minor clone (bottom panel).

As an extended test, we next considered three additional tumour types: Wilms tumour, ATRT, and Ewing’s sarcoma. Unlike RCC and neuroblastoma, CopyKAT and alleleIntegrator were both able to correctly identify leucocytes and endothelial cells as not cancer derived (Fig. 3a, Supplementary Fig. 5). However, CopyKAT incorrectly identifies the majority of Wilms tumour cells as normal (Fig. 3a). This is likely driven by the heterogeneous nature of Wilms tumour, which produces multiple populations of transcriptionally and histologically distinct cancer cells. Next, we compared each method’s copy-number profile to the ground truth, calculating the sensitivity and specificity with which each method identified neutral/altered genomic regions (Fig. 3b, Supplementary Fig. 3). We found similar levels of performance for both expression-based methods, both of which performed poorly compared to allelic ratios (Fig. 3b). We next asked how clearly regions of gain and loss could be separated from one another by each of the three methods. Looking across all samples, we found that distribution of expression values for regions with no change, copy-number gain, and copy-number loss strongly overlapped (Fig. 3c, Supplementary Fig. 3). By contrast, each of these three types of region produced clearly separated peaks in the distribution of allelic ratios (Fig. 3c). Across our tests, we found both expression-based copy-number callers to perform similarly and to have highly correlated outputs (Supplementary Fig. 6). Therefore, the properties of expression-based copy-number callers are likely general, not specific to inferCNV and CopyKAT.

Beyond distinguishing cancer and normal cells, the high precision of copy-number genotyping by allelic ratios may lend itself to the identification of minor cancer cell populations (subclones) defined by copy-number aberrations. We investigated cancer subclone identification in a neuroblastoma (PD46693) that harboured a minor clone, comprising ~30% of cells, defined by copy-number neutral loss of heterozygosity of chromosome 4. AlleleIntegrator identified 389/1282 sub-clonal cells with a posterior probability of more than 99% (Fig. 3d). These cells are transcriptionally extremely similar, with only 95 genes and 7 transcription factors significantly differentially expressed between the major and minor clones (Supplementary Fig. 7, Supplementary Tables 2, 3). Amongst these genes were neuroblastoma-associated genes *NTRK1, BCL11A*, *TH* and *CHGB*, as well as *HMX1*, a transcription factor on chromosome 4 that is a master regulator of neural crest development. Although we would not claim that these genes collectively or individually are the definitive target of the sub-clonal copy-number change, this analysis illustrates the power of our approach in deriving functional hypotheses about copy-number changes. This is particularly pertinent in neuroblastoma, where clinical risk is defined by segmental copy-number changes that remain functionally cryptic^12^.

## Discussion

We have shown that allelic imbalances that represent cancer-defining somatic copy-number changes can precisely identify single cancer cell transcriptomes. A prerequisite of this approach, that limits its application, is the presence (and knowledge) of somatic copy-number changes. We consider the main utility of our approach to lie in corroborating or refuting claims of novel cancer cell types and for investigating the functional consequences of sub-clonal copy-number changes. We found expression-based copy-number detection tools to produce highly correlated results, suggesting that the limitations are general to the approach, not specific to the implementation. Where direct nucleotide interrogation is not feasible, the expression of marker genes and detection of average shifts in expression with tools such as CopyKAT, may still provide a reasonable basis for indirectly inferring which single cell transcriptomes possess the somatic cancer genotype. However, our observations caution against identifying novel cancer cell types through such approaches alone, without direct interrogation of underlying nucleotide sequence. Accordingly, our findings suggest that it may be warranted to reappraise recent claims of novel cell types in a variety of cancers, such as neuroblastoma, that were solely based on expression-based cancer cell identification.

## Methods

### Identifying cancer cells using allelic ratio

To identify cancer cell transcriptomes, we used a bayesian statistical framework^8,9^, implemented in an R package, alleleIntegrator. The calling of cancer cell transcriptomes has four steps (Fig. 1a):

1. Call copy-number changes and heterozygous SNPs.
2. Phase heterozygous SNPs within regions with altered copy-number using tumour DNA.
3. Count reads supporting the major/minor allele in each copy-number segment/transcriptome.
4. Calculate posterior probability of the cancer and normal genotype.

The precise step-by-step implementation is contained in the provided code and a detailed description of each step is provided below.

#### Calling heterozygous SNPs and copy-number changes

We identified copy-number (CN) states using Battenberg^13^ applied to whole genome sequencing of tumour DNA. SNPs were called using bcftools mpileup/call to find sites with reads supporting two alleles and a BAF between 0.2 and 0.8. Sites inconsistent with heterozygosity were excluded using a binomial test with 5% FDR^14^. Alternatively, CN states and heterozygous SNP locations can be provided using alternate methods.

#### Phasing heterozygous SNPs in copy-number region(s)

Using alleleCount (https://github.com/cancerit/alleleCount), we counted reads supporting each allele in tumour DNA in regions of uneven CN (i.e. where the number of maternal/paternal copies differ). The reference (or alternate) allele was assigned to the minor allele when the BAF was greater than (or less than) 0.5. Sites not significantly different from 0.5 (binomial test, 5% FDR^14^) were excluded.

#### Counting reads by allele in each transcriptome

At each phased SNP, we calculated the counts supporting the major and minor allele for each transcriptome using alleleCount in 10X mode (−x flag). These were summed by segment/transcriptome, producing a table of major and minor allele counts for each transcriptome and copy-number segment.

#### Calculating posterior probability of cancer genotype

We aimed to compare two possibilities: that the cell contains the cancer genotype or the normal genotype. To this end, we constructed a model that accounts for the major known error processes and properties of transcription: errors can alter the observed allele, transcription occurs in bursts, and transcription can exhibit allelic bias. We used a negative binomial likelihood, where the overdispersion captures extra variability due to transcriptional bursts.

We first filter out SNPs that: are imprinted (i.e. only ever express one allele), are not intronic or exonic, or have zero coverage. This filtering is most accurate when cells with the normal genotype can be specified (e.g. leucocytes in a solid tissue tumour). We also exclude genes known to display complex allele-specific expression (ASE), specifically, *HLA* and *HB* genes.

We specify a site-specific error rate of 0.01 for exonic reads and 0.05 for intronic reads, calibrated by counting non-reference reads at sites homozygous for the reference.

After filtering, we calculate the posterior probability of allele-specific expression in normal cells for each gene, using a beta distribution prior with mean 0.5 and spread set manually or to the best fit value of highly expressed genes (default genes > 400 counts). Where normal cells are not given, both alleles are considered equally likely.

Next, we calculate the maximum likelihood value of the beta-binomial overdispersion from normal cells using the error rate and ASE values derived above. We optionally marginalise this estimate over the ASE posterior distribution, although we find this step makes no difference to the final estimate. Where normal calls are not given, the overdispersion is set manually or the best fit is calculated across all cells. Including non-normal cells increases the overdispersion, making downstream calls of which cells are cancer-derived more conservative.

The expected allelic ratio at a SNP s is then given by

1 $${r}_{s}(f)\,=\, \bigg (\frac{f{\rho }_{s}}{f{\rho }_{s}\,+\,(1\,-\,f)(1\,-\,{\rho }_{s})} \bigg )(1\,-\,2{\epsilon }_{s})\,+\,{\epsilon }_{s}$$

where *f* is the number of major copies of the segment as a fraction of the total (i.e. 0.5 for diploid, 2/3 for a gain of one copy, 1 for the loss of one copy), ***ρ*** is the ASE ratio (i.e. 0.5 for no ASE) and ε is the site-specific error rate.

Using this ratio, the likelihood of a region R, having a major allele fraction f is given by,

2 $${P}_{R}\left({data} | f\right)\,=\,{\varPi }_{s\in R}\frac{{m}_{s}\,+\,{n}_{s}}{{m}_{s}}\,\frac{B\big({m}_{s}\,+\,{r}_{s}(f)\big(\frac{1\,-\,\phi }{\phi }\big),{n}_{s}\,+\, \big(1\,-\,{r}_{s}(f)\big)\big(\frac{1\,-\,\phi }{\phi }\big)\big)}{B\big({r}_{s}(f)\big(\frac{1\,-\,\phi }{\phi }\big),\big(1\,-\,{r}_{s}(f)\big)\big(\frac{1\,-\,\phi }{\phi }\big)\big)}$$

where ${m}_{s}$ and ${n}_{s}$ are the number of counts at SNP s from the major and minor allele respectively, $\phi$ is the previously estimated overdispersion, and B is the standard beta function. Note that the above is just a beta-binomial likelihood, which has been re-parameterise in terms of the mean probability of the beta distribution (r) and the variance of the beta distribution ($\phi$). The sum is taken across all SNPs that lie within the region R.

To get the total likelihood that each cell is cancer derived, we then take the product across all regions with copy-number change, setting $f$ equal to the implied copy-number fraction in each region, ${a}_{R}$. That is,

3 $$P({data}{{{{{\rm{|}}}}}}{cancer})\,=\,{\varPi }_{R}{P}_{R}({data}{{{{{\rm{|}}}}}}{f\,=\,a}_{R})$$

where ${a}_{R}\,=\,1$ in regions of loss of heterozygosity, ${a}_{R}\,=\,2/3$ in regions where 1 copy is gained, etc. By contrast, the likelihood for the cell being normal is given by setting $f\,=\,0.5\forall R$, that is

4 $$P({data}{{{{{\rm{|}}}}}}{normal})\,=\,{\varPi }_{R}{P}_{R}({data}{{{{{\rm{|}}}}}}f\,=\,0.5)$$

Finally, the posterior probability of a cell being cancer is calculated assuming a flat prior as,

5 $$P({cancer}{{{{{\rm{|}}}}}}{data})\,=\,\frac{P({data}{{{{{\rm{|}}}}}}{cancer})}{P({data}{{{{{\rm{|}}}}}}{cancer})\,+\,P({data}{{{{{\rm{|}}}}}}{normal})}$$

Each cell is then assigned as cancer where $P({cancer|data})$ exceeds 0.99, normal where it is <0.01, and unassigned otherwise.

### Statistics and reproducibility

Statistical analysis was performed as described elsewhere in the methods. The selection of samples for benchmarking purposes was chose to represent a coverage of a broad range of different cancer types, with biological replicates (different cancers of the same type) and technical replicates (multiple single cell transcriptomics reactions from the same individual) were generated wherever possible.

### Ethics approval

Human tumour tissues were collected through studies approved by UK NHS research ethics committees. Patients or guardians provided informed written consent for participation in this study as stipulated by the study protocols. This study has the reference NHS National Research Ethics Service reference 16/EE/0394 (paediatric tissues).

### 10X single cell sequencing of fresh tissue and bulk sequencing of DNA

Fresh tissues were processed to generate single suspensions for processing on the Chromium 10X controller (V2/3 3′ chemistry), as previously described^8^. Libraries were produced according to the manufacturer’s instructions and sequenced on an Illumina HiSeq4000 device. Sequencing of bulk DNA was performed as previously described^8^.

### Data QC, clustering, and visualisation

We used R (v4.0.4) and Seurat (v.4.0.3) for these analyses. Cells with <200 genes, <600 UMIs, mitochondrial fraction exceeding 20% (30% for renal cell carcinoma (RCC) normal tissue), or Scrublet^15^ doublet score >0.5 were excluded. High resolution clusters (resolution = 10) with >50% cells failing QC were also excluded.

Data were log normalised and scaled, and principal components were calculated using highly variable genes using the standard Seurat workflow. Louvain clustering was performed with resolution 1 and a uniform manifold approximation and projection (UMAP) calculated, with the number of principal components used for each dataset as follows: 25 for RCC, 30 for Ewing’s sarcoma, 40 for Wilms tumour, 50 for atypical teratoid rhabdoid tumour (AT/RT), and 55 for neuroblastoma (NB). Finally, cells from RCC and NB datasets were labelled using the published annotation; and leucocytes, endothelium, mesenchyme (in NB only), proximal tubular cells (in RCC only), and tumour cells were retained. Annotation of Wilms tumour, Ewing’s sarcoma and AT/RT datasets was performed based on expression of known genetic markers, curated from literature, for different cell types, including tumour populations.

### Coverage of point mutations and heterozygous SNPs

For all samples, heterozygous SNPs were identified (as described above) and point mutations were called against the GRCh37d5 reference as previously described^8,9^. Coordinates were lifted over to GRCh38 and counts covering point mutations and SNPs were calculated for each transcriptome using allele counter.

### Calling copy-number aberrations

Clonal and sub-clonal copy-number profiles were determined using Battenberg^13^ (v2.2.5). Segments shorter than 1 Mb or 10% of the chromosome were removed as likely artifacts. Chromosomes were set to the same state where ≥90% had a particular change and gaps filled where consecutive segments had the same copy-number state and were <1 Mb apart. Sub-clonal copy-number segments were defined as those with a second copy-number state detected in a smaller fraction of tumour cells (≥10% but <50%) and are longer than 20 Mb.

### Evaluating accuracy of transcriptome classification

inferCNV^1^ (1.6.0) and CopyKAT^2^ (v1.0.4) were run with default parameters per-sample using cellranger filtered counts and 100% of leucocytes specified as normal. Both methods generate expression profiles on a log scale for informative cells within each sample. In addition, CopyKAT also classified these cells as being diploid, aneuploid, or uncalled. For each sample, expression ratio per 5 Mb window was averaged by cell type. A range of thresholds were used to quantify copy-number call accuracy and construct a receiver operating characteristic (ROC) curve. To assess the correlation between average expression ratios calculated by CopyKAT and inferCNV, a Pearson correlation coefficient was calculated using R.

To visualise the allelic ratio in each sample, allele-specific counts were aggregated by cell type into bins chosen such that each bin contained at least 500 counts.

### Analysis of PD46693 subclones

Cells with posterior probability >0.99 of loss of heterozygosity of chromosome 4 in PD46693 were assigned to the subclone, those with posterior probability <0.01 were assigned to the major clone, and all others were called ambiguous.

Differential gene expression was performed using negative binomial regression in DESeq2^16^, treating cells in the major/minor clone as replicates and removing genes with ≤10 reads. We separately tested all genes and just transcription factors for significance, using a multiple hypothesis corrected^4^ *p* value cut-off of 0.01.

### Reporting summary

Further information on research design is available in the Nature Research Reporting Summary linked to this article.

## Supplementary information

Supplementary Information

Description of Additional Supplementary Files

Supplementary Data 1

Supplementary Data 2

Reporting Summary

## Data Availability

WGS-derived clonal and sub-clonal copy-number profile for individual samples, identified by Battenberg^13^, can be found in Supplementary Data 1. Previously published data was obtained for renal cell carcinoma^8^, neuroblastoma^9^, and Wilms tumour^10^. Newly generated data for atypical teratoid rhabdoid tumour (AT/RT) and Ewing’s sarcoma has been deposited in the EGA under accession code EGAD00001009005. Numerical source data underlying figures can be found in Supplementary Data 2.

## Code availability

The R package, alleleIntegrator, is available at https://github.com/constantAmateur/alleleIntegrator. All code used to reproduce the analysis and figures described in this paper is available at https://github.com/mitrinh1/scGenotyping. The exact versions of both the R package and analysis code used for this paper are also available from zenodo^17,18^.
