# Supplementary material for: Precise identification of cancer cells from allelic imbalances in single cell transcriptomes
Source: Commun Biol. 2022 Sep 7;5:884. doi: 10.1038/s42003-022-03808-9 (PMC9452529; doi:10.1038/s42003-022-03808-9)
Supplement: Supplementary file 1 — Supplementary Information [file 42003_2022_3808_MOESM1_ESM.pdf]

Supplementary Information for

Precise identification of cancer cells from allelic imbalances in single cell transcriptomes

Mi K. Trinh[1], Clarissa N. Pacyna[1], Gerda Kildisiute[1], Christine Thevanesan[3], Alice Piapi[3], Kirsty Ambridge[1], Nathaniel D. Anderson[1], Eleonora Khabirova[1], Elena Prigmore[1], Karin Straathof[3], Sam Behjati*[1,2,4], Matthew D. Young*[1]

[1]Wellcome Sanger Institute, Wellcome Genome Campus, Hinxton, Cambridge CB10 1SA, UK.
[2]Cambridge University Hospitals NHS Foundation Trust, Cambridge, CB2 0QQ, UK.
[3]University College London Great Ormond Street Biomedical Research Centre, London, WC1E 6BT, UK.
[4]Department of Paediatrics, University of Cambridge; Cambridge, UK.

*Correspondence: Matthew D. Young - my4@sanger.ac.uk, Sam Behjati - sb31@sanger.ac.uk

Supplementary Figures

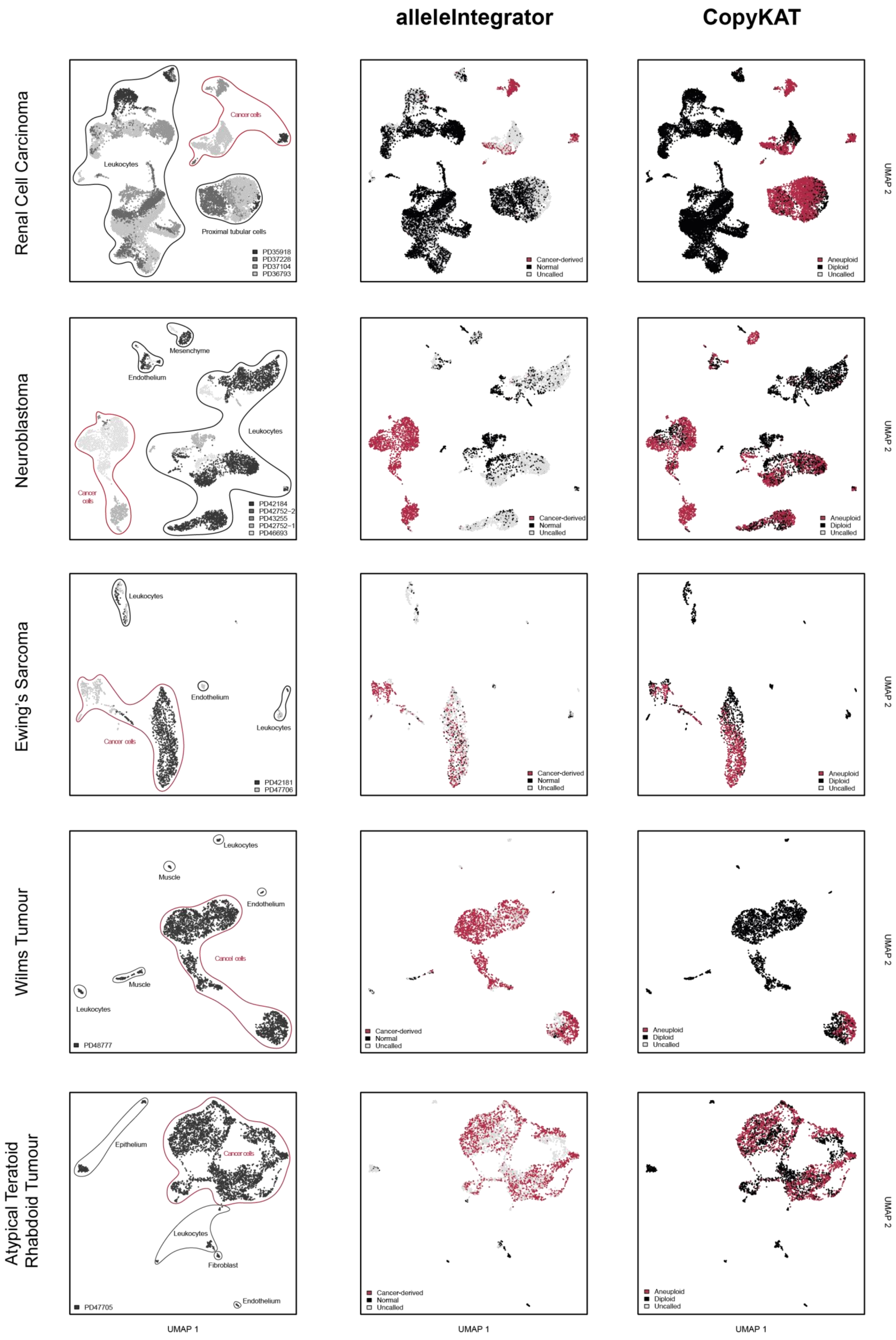
alleleIntegrator
CopyKAT
Renal Cell Carcinoma
Leukocytes
Cancer cells
Proximal tubular cells
PD35918
PD37228
PD37104
PD36793
Cancer-derived
Normal
Uncalled
Aneuploid
Diploid
Uncalled
Neuroblastoma
Mesenchyme
Endothelium
Leukocytes
Cancer cells
PD42184
PD42752−2
PD43255
PD42752−1
PD46693
Ewing's Sarcoma
Leukocytes
Endothelium
Cancer cells
PD42181
PD47706
Wilms Tumour
Leukocytes
Muscle
Endothelium
Cancel cells
PD48777
Atypical Teratoid Rhabdoid Tumour
Epithelium
Cancer cells
Leukocytes
Fibroblast
Endothelium
PD47705
UMAP 1
UMAP 2

Supplementary Figure 1 – UMAPs of patients and cancer transcriptome calls

Two-dimensional visualisation (using UMAP) of single cell transcriptomes from five different tumour types (rows), showing cell type annotation (left), calls made by alleleIntegrator (middle), and by CopyKAT (right).

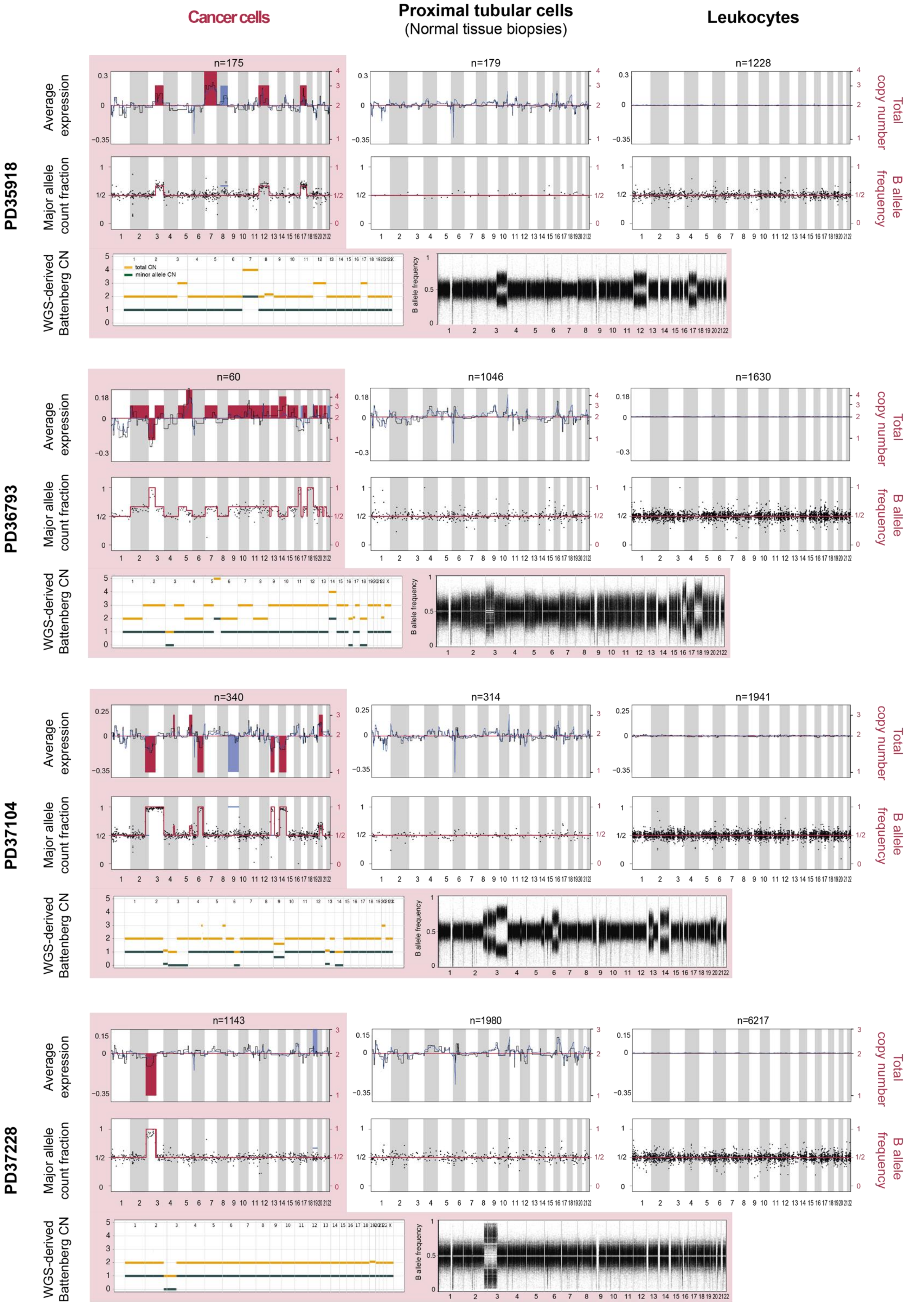

Cancer cells
Proximal tubular cells
(Normal tissue biopsies)
Leukocytes
PD35918
PD36793
PD37104
PD37228
Average expression
Major allele count fraction
WGS-derived Battenberg CN
Total copy number
B allele frequency
n=175
n=179
n=1228
n=60
n=1046
n=1630
n=340
n=314
n=1941
n=1143
n=1980
n=6217
total CN
minor allele CN
B allele frequency

Supplementary Figure 2 – Copy number profiles of renal cell carcinomas

Copy number profile for 5 renal cell carcinoma samples from normalised averaged expression ratio (top panels, solid black line for CopyKAT, solid blue line for inferCNV) and allelic ratio (middle panel, one dot per bin with ~500 reads), with ground truth from whole genome sequencing (WGS) (red, arbitrary scale in top panel). Sub-clonal copy number changes shown in blue.

Bottom panel of each sample shows the WGS-defined copy number changes by Battenberg (green line represents the minor allele copy number state, yellow line represents total copy number state), and the corresponding WGS–defined B allele frequency.

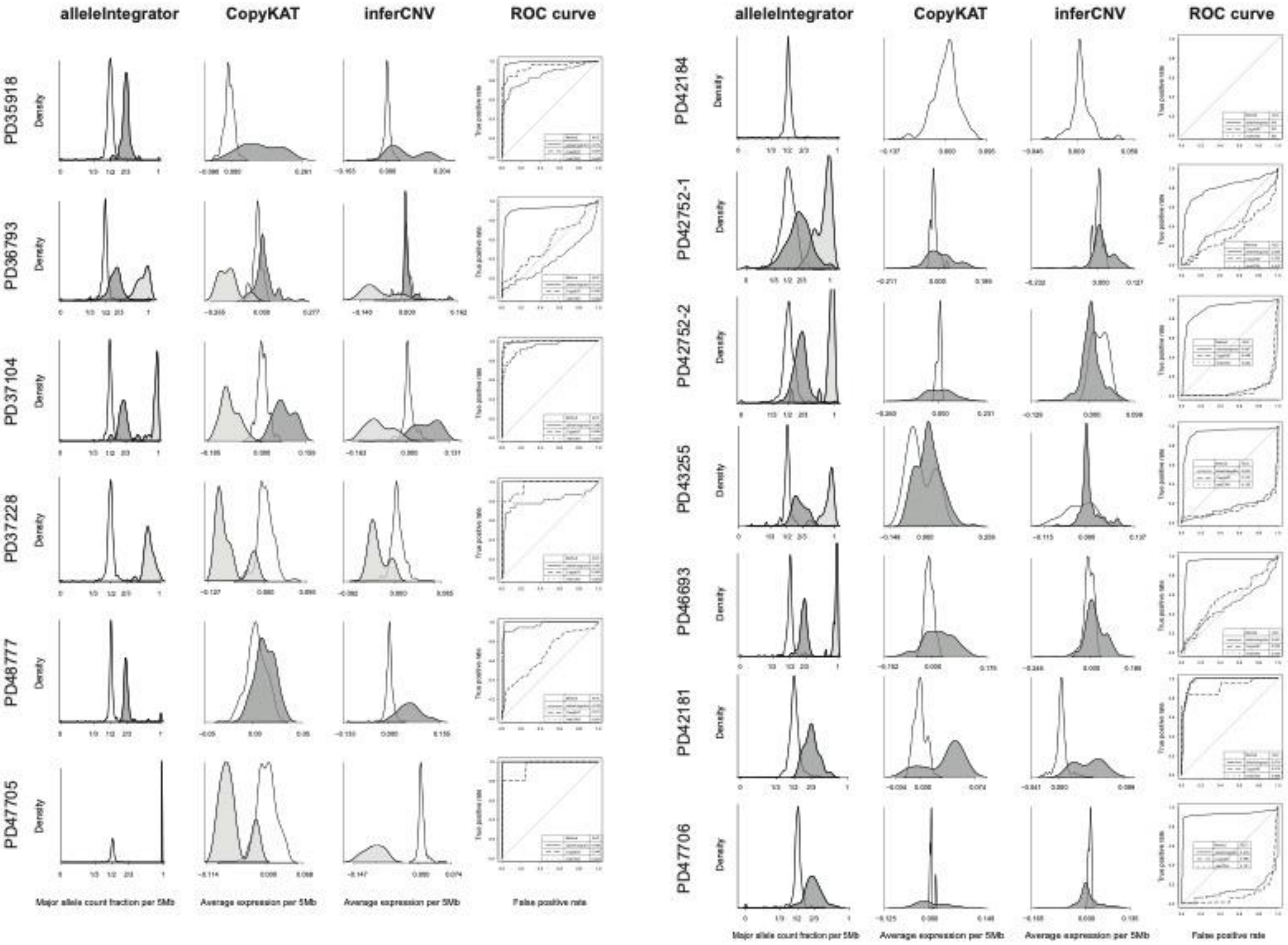
alleleIntegrator
CopyKAT
inferCNV
ROC curve
PD35918
PD36793
PD37104
PD37228
PD48777
PD47705
PD42184
PD42752-1
PD42752-2
PD43255
PD46693
PD42181
PD47706
Density
True positive rate
Major allele count fraction per 5Mb
Average expression per 5Mb
Average expression per 5Mb
False positive rate

Supplementary Figure 3 – Copy number profile evaluation by individual

For each individual (rows), the distribution of allelic ratios (left column) and expression ratios (middle columns) in 5 megabase bins, generated using alleleIntegrator, CopyKAT, and inferCNV, sequentially from the left. Each plot is colour coded by regions with copy number gains (dark shading), losses (light shading), or no change (white). The rightmost column displays a receiver operating characteristic (ROC) curve showing the sensitivity and specificity with which each method recovers the ground truth.

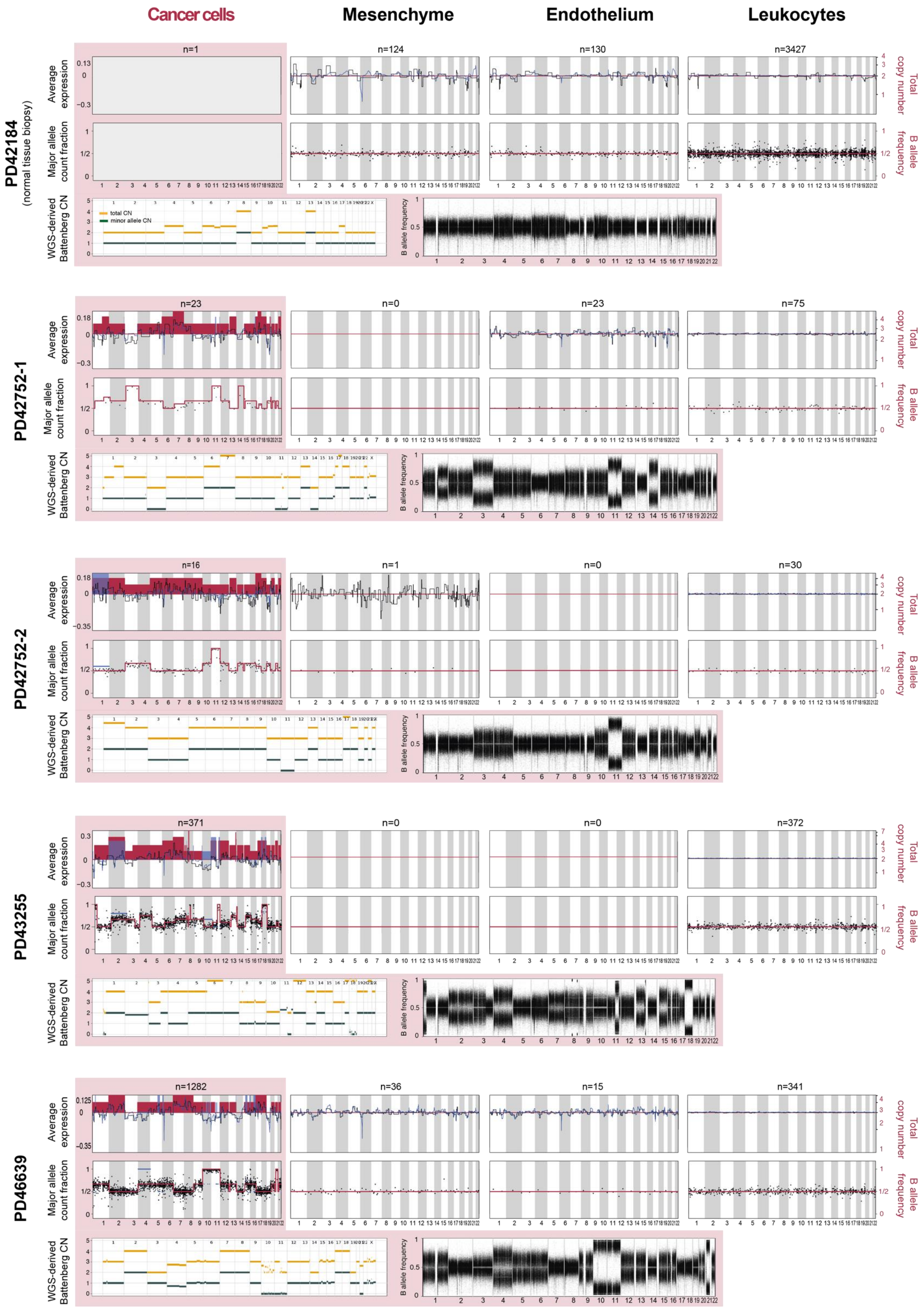

Cancer cells
Mesenchyme
Endothelium
Leukocytes
PD42184
(normal tissue biopsy)
PD42752-1
PD42752-2
PD43255
PD46639
n=1
n=124
n=130
n=3427
n=23
n=0
n=23
n=75
n=16
n=1
n=0
n=30
n=371
n=0
n=0
n=372
n=1282
n=36
n=15
n=341
Average expression
Major allele count fraction
WGS-derived Battenberg CN
total CN
minor allele CN
B allele frequency
Total copy number
B allele frequency

Supplementary Figure 4 – Copy number profiles of neuroblastomas

As per **Supplementary Figure 2** but for neuroblastoma.

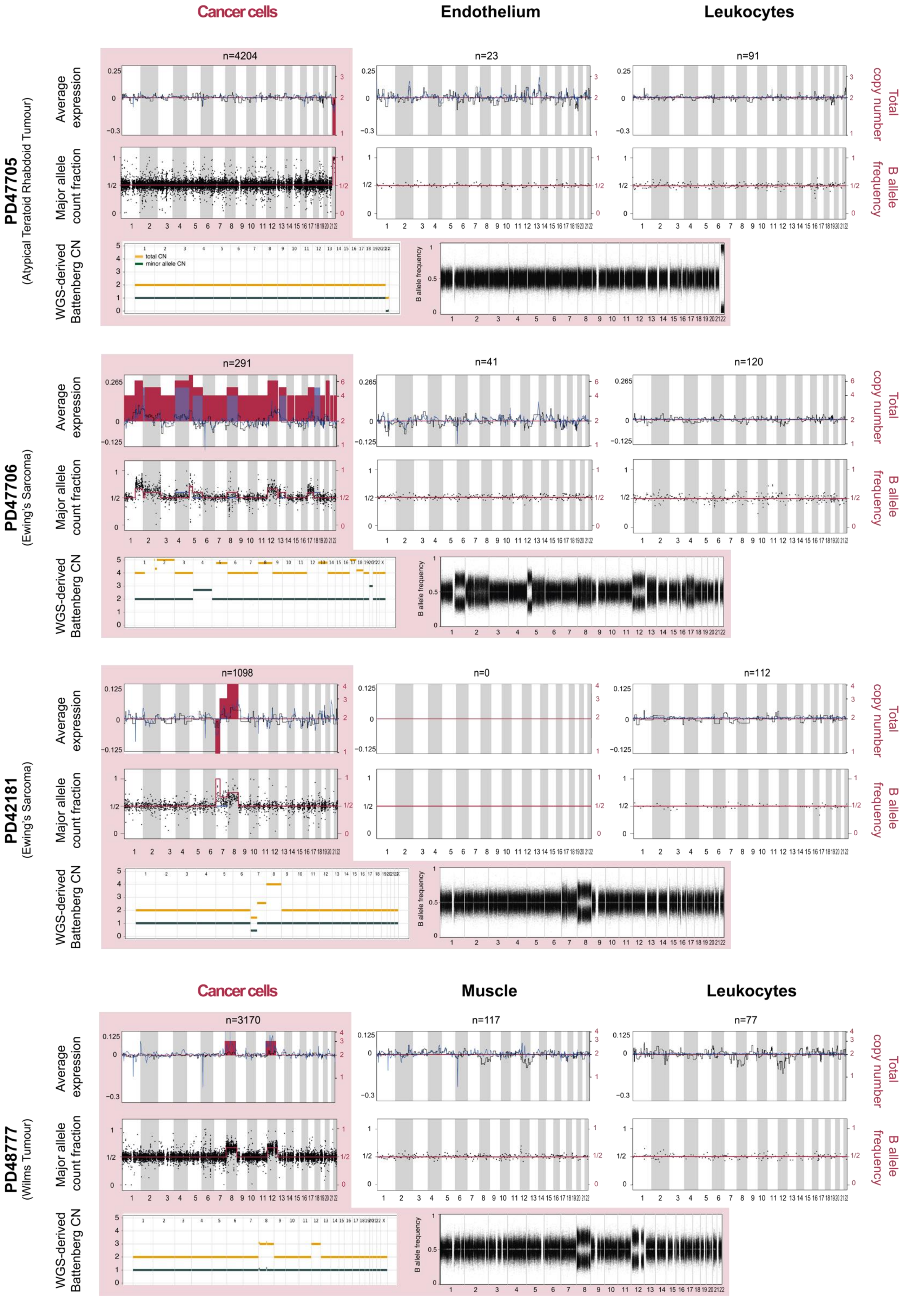
Cancer cells
Endothelium
Leukocytes
PD47705
(Atypical Teratoid Rhabdoid Tumour)
n=4204
n=23
n=91
Average expression
Major allele count fraction
WGS-derived Battenberg CN
total CN
minor allele CN
B allele frequency
Total copy number
B allele frequency
PD47706
(Ewing's Sarcoma)
n=291
n=41
n=120
PD42181
(Ewing's Sarcoma)
n=1098
n=0
n=112
Cancer cells
Muscle
Leukocytes
PD48777
(Wilms Tumour)
n=3170
n=117
n=77

Supplementary Figure 5 – Copy number profiles of Wilms tumour, Ewing's, and AT/RT

As per **Supplementary Figure 2** but for Wilms tumour, Ewing's sarcoma, and atypical teratoid rhabdoid tumour.

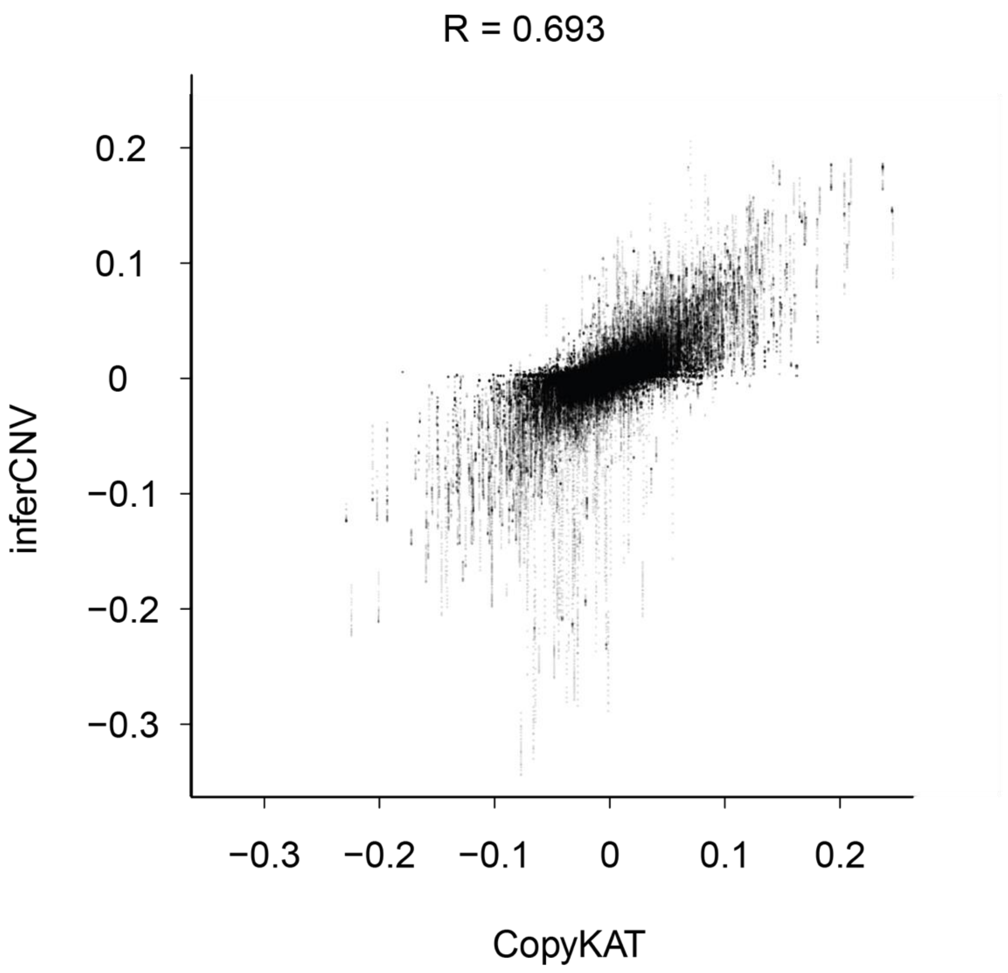

Supplementary Figure 6 – Correlation of inferCNV and CopyKAT output

Scatter plot showing the relationship between the CopyKAT (x-axis) and inferCNV (y-axis) output values, averaged in 5 megabase bins. The value in the top-left gives the Pearson correlation coefficient.

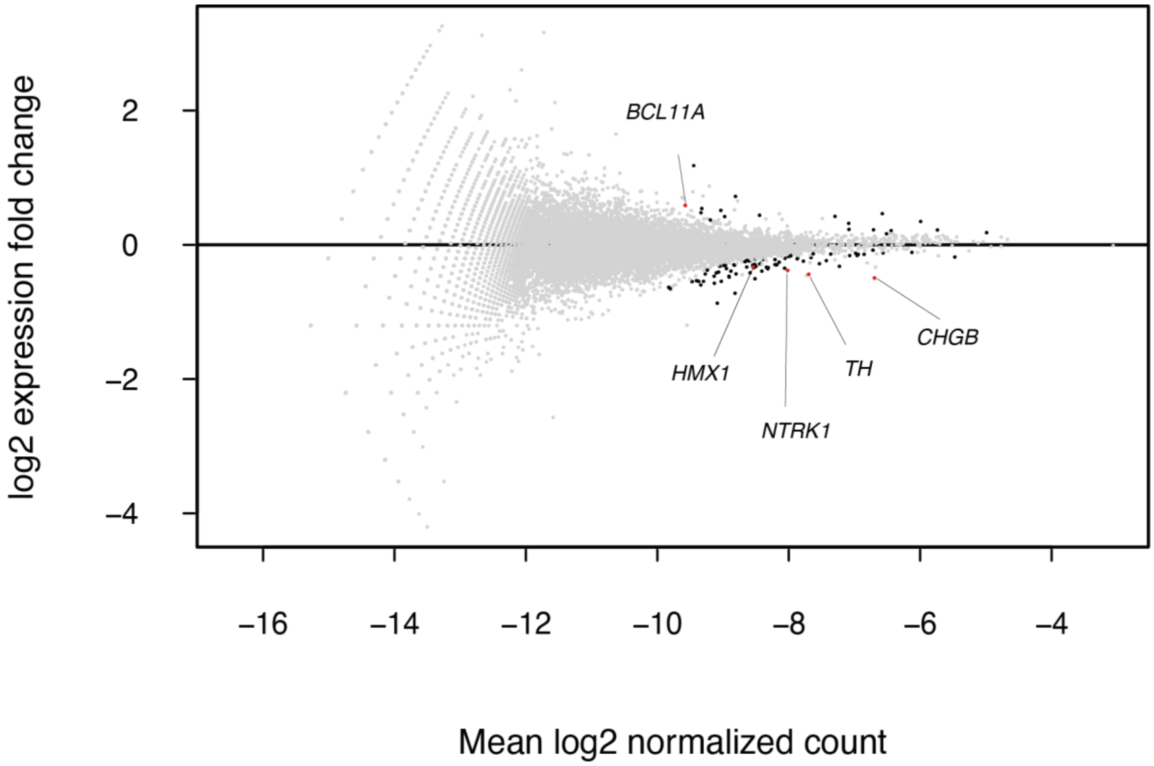

Supplementary Figure 7 – Differential expression between PD46693 major and minor clones

Log fold change (y-axis) for each gene (points) by average normalised expression (x-axis), with differentially expressed genes/transcription factors shown in black.

Supplementary Tables

Patient ID	Disease type	Biopsy type	Source
PD35918	Renal cell carcinoma	Normal	Young, M. D. et al., 2018 [1]
PD35918	Renal cell carcinoma	Tumour	Young, M. D. et al., 2018 [1]
PD37228	Renal cell carcinoma	Normal	Young, M. D. et al., 2018 [1]
PD37228	Renal cell carcinoma	Tumour	Young, M. D. et al., 2018 [1]
PD37104	Renal cell carcinoma	Normal	Young, M. D. et al., 2018 [1]
PD37104	Renal cell carcinoma	Tumour	Young, M. D. et al., 2018 [1]
PD36793	Renal cell carcinoma	Normal	Young, M. D. et al., 2018 [1]
PD36793	Renal cell carcinoma	Tumour	Young, M. D. et al., 2018 [1]
PD42184	Neuroblastoma	Normal	Kildisiute, G. et al., 2021 [2]
PD42752-1	Neuroblastoma	Tumour	Kildisiute, G. et al., 2021 [2]
PD42752-2	Neuroblastoma	Tumour	Kildisiute, G. et al., 2021 [2]
PD46693	Neuroblastoma	Tumour	Kildisiute, G. et al., 2021 [2]
PD43255	Neuroblastoma	Tumour	Kildisiute, G. et al., 2021 [2]
PD48777	Wilms tumour	Tumour	Young, M. D. et al., 2021 [3]
PD47705	Atypical teratoid rhabdoid tumour	Tumour	This study, EGAD00001009005
PD42181	Ewing's sarcoma	Tumour	This study, EGAD00001009005
PD47706	Ewing's sarcoma	Tumour	This study, EGAD00001009005

Supplementary Table 1 – Sample manifest

A table linking sample IDs to their metadata.

gene	baseMean	log2FoldChange	lfcSE	stat	pvalue	padj	sig
HES4	0.8253738	-0.4477104	0.1117724	-4.005553	6.19E-05	0.0060718	TRUE
HMGN2	5.8759454	-0.3009108	0.0839695	-3.583573	3.39E-04	0.0169118	TRUE
NUDC	1.4732091	0.2489479	0.0723027	3.4431327	5.75E-04	0.0245341	TRUE
TMEM59	2.637107	0.200375	0.0621226	3.2254757	0.0012576	0.0417138	TRUE
RPL5	8.6186412	-0.2036441	0.0532526	-3.8241191	1.31E-04	0.0087812	TRUE
CKS1B	1.3903648	-0.4285162	0.1223202	-3.5032337	4.60E-04	0.020197	TRUE
NTRK1	2.147035	0.4380721	0.0955862	4.5830036	4.58E-06	0.0013494	TRUE
RGS4	0.8592059	0.5968772	0.1630565	3.6605549	2.52E-04	0.0148183	TRUE
ATP1B1	2.9103937	0.2895028	0.0805064	3.5960218	3.23E-04	0.0167372	TRUE
BCL11A	0.6173239	-0.5659022	0.1442805	-3.9222359	8.77E-05	0.0071744	TRUE
COX5B	6.1128152	0.1568272	0.0405295	3.8694593	1.09E-04	0.0080281	TRUE
RND3	0.9053124	-0.409618	0.1259249	-3.2528743	0.0011424	0.0395688	TRUE
ORMDL1	1.3591736	0.2492442	0.0782012	3.1872196	0.0014365	0.045473	TRUE
TMEFF2	1.3631924	0.3695228	0.101376	3.6450712	2.67E-04	0.015431	TRUE
ITM2C	1.8509359	0.3274491	0.0791724	4.1359001	3.54E-05	0.0041934	TRUE
ECEL1	1.5043682	0.4077567	0.0989572	4.1205378	3.78E-05	0.00428	TRUE
RAMP1	5.7444115	0.1716878	0.0529584	3.2419371	0.0011872	0.040641	TRUE
GATA2	0.8563014	0.4728011	0.1330183	3.5544059	3.79E-04	0.0182834	TRUE
HMX1	1.1620017	0.3751426	0.1113263	3.3697572	7.52E-04	0.0307625	TRUE
CCNI	5.8631001	-0.2307558	0.0586053	-3.9374556	8.24E-05	0.0069268	TRUE
HSD17B11	0.6296871	-0.3977966	0.1135235	-3.5040912	4.58E-04	0.020197	TRUE
SPP1	1.3749537	-0.8205298	0.1849113	-4.4374241	9.10E-06	0.0017954	TRUE
NAP1L5	0.6963927	0.8258064	0.0969394	8.5187891	1.61E-17	4.75E-14	TRUE
GRIA2	0.7755683	0.4945109	0.1237505	3.99603	6.44E-05	0.0061172	TRUE
TENM3	0.8640558	-0.5113776	0.1308792	-3.9072497	9.34E-05	0.0074278	TRUE
BASP1	10.403841	-0.1858031	0.0484627	-3.8339389	1.26E-04	0.008634	TRUE
EDIL3	0.5796694	0.5548026	0.1250569	4.4364006	9.15E-06	0.0017954	TRUE
PAM	0.4459023	0.4951133	0.1484599	3.334996	8.53E-04	0.0334309	TRUE
CAMK4	0.7884342	0.3390501	0.1052747	3.2206242	0.0012791	0.0418414	TRUE
GFRA3	1.7573593	0.2547571	0.0736544	3.4588169	5.43E-04	0.0234894	TRUE
NDFIP1	3.0502333	0.2244127	0.0581966	3.8561166	1.15E-04	0.0080752	TRUE
CANX	1.0366419	0.3011137	0.0925487	3.2535697	0.0011396	0.0395688	TRUE
HIST1H4C	8.6883692	-0.4200807	0.1186053	-3.5418384	3.97E-04	0.0185682	TRUE
TUBB	20.290442	-0.1934457	0.0501003	-3.8611719	1.13E-04	0.0080752	TRUE
HSPA1A	1.1411987	0.5349498	0.1471919	3.6343701	2.79E-04	0.0157765	TRUE
HSPA1B	0.628758	0.555954	0.1708593	3.2538714	0.0011384	0.0395688	TRUE
CDC5L	0.797059	0.4361822	0.1017803	4.2855281	1.82E-05	0.0026835	TRUE
RP3-525N10.2	1.8252654	0.3073796	0.0951067	3.2319453	0.0012295	0.0416053	TRUE
SEC63	0.9279419	0.4061351	0.0940798	4.316919	1.58E-05	0.0024516	TRUE
NCOA7	1.6574015	0.365465	0.0860142	4.2488916	2.15E-05	0.0028753	TRUE
NDUFA4	6.9138012	0.159141	0.0422514	3.7665298	1.66E-04	0.0105941	TRUE
RAMP3	0.6781477	0.5602232	0.1058501	5.2926112	1.21E-07	7.10E-05	TRUE
DDC	1.1975783	0.3001319	0.0943018	3.182674	0.0014592	0.0457015	TRUE
VSTM2A	1.9723473	0.3165741	0.0788356	4.015622	5.93E-05	0.0060718	TRUE
HSPB1	6.4576771	0.2247478	0.0577539	3.891473	9.96E-05	0.0077193	TRUE
NRCAM	0.7717933	0.3972158	0.1102222	3.6037724	3.14E-04	0.0167372	TRUE
EXOC4	1.6527206	0.4501881	0.1140501	3.9472843	7.90E-05	0.0068442	TRUE

TMSB4X	46.249748	-0.1504748	0.0421414	-3.5707114	3.56E-04	0.0174684	TRUE
PCSK1N	9.4854987	0.1586586	0.0484032	3.2778506	0.001046	0.0384931	TRUE
DUSP26	1.1813755	0.3773431	0.0871979	4.3274326	1.51E-05	0.0024516	TRUE
NDUFB9	4.1439026	0.1694863	0.044991	3.7671164	1.65E-04	0.0105941	TRUE
VCP	0.9074799	0.3200255	0.0901912	3.548303	3.88E-04	0.0184105	TRUE
GOLM1	1.0384772	0.3246282	0.0901599	3.6005833	3.18E-04	0.0167372	TRUE
SPTAN1	1.4877743	0.3662087	0.0822526	4.4522449	8.50E-06	0.0017954	TRUE
DBH	3.4470086	0.3259419	0.078052	4.1759584	2.97E-05	0.0037982	TRUE
TH	2.9677261	0.4255725	0.0754896	5.6374969	1.73E-08	1.69E-05	TRUE
CD44	1.2290094	0.4767816	0.0963983	4.9459548	7.58E-07	3.19E-04	TRUE
FKBP2	1.2098143	0.2581147	0.0789477	3.2694401	0.0010776	0.0391663	TRUE
CADM1	2.2422979	0.2486676	0.0669907	3.7119709	2.06E-04	0.0128817	TRUE
SLC18A2	0.6418574	0.5022565	0.1294599	3.8796295	1.05E-04	0.0078971	TRUE
RGS10	0.6119829	-0.9576624	0.1485281	-6.4476863	1.14E-10	1.67E-07	TRUE
CD9	1.5324888	0.3859138	0.0976675	3.9513019	7.77E-05	0.0068442	TRUE
NELL2	0.6436465	0.4259095	0.1158716	3.6757021	2.37E-04	0.014548	TRUE
TUBA1B	15.310965	-0.3572856	0.0728195	-4.9064578	9.27E-07	3.41E-04	TRUE
PRPH	4.3624368	-0.3359695	0.0833599	-4.0303502	5.57E-05	0.0060718	TRUE
FAIM2	1.892397	0.3262801	0.0676517	4.8229435	1.41E-06	4.63E-04	TRUE
HSP90B1	1.4203509	0.406519	0.1020029	3.9853661	6.74E-05	0.0061986	TRUE
COX6A1	8.1570732	0.1169433	0.0364978	3.2041205	0.0013548	0.0433522	TRUE
MYCBP2	0.9100619	0.3393452	0.1040627	3.2609691	0.0011103	0.0395688	TRUE
DAD1	0.83115	0.3596553	0.0869939	4.1342605	3.56E-05	0.0041934	TRUE
PSME2	0.60898	0.3716209	0.1034003	3.5940022	3.26E-04	0.0167372	TRUE
EGLN3	0.3995463	0.5068218	0.148344	3.4165314	6.34E-04	0.0264981	TRUE
CHGA	1.9369055	0.4151369	0.0956949	4.3381301	1.44E-05	0.0024516	TRUE
IFI27	0.7784338	0.6041944	0.1723234	3.5061648	4.55E-04	0.020197	TRUE
PPP2R5C	0.7374246	0.4364171	0.108882	4.0081654	6.12E-05	0.0060718	TRUE
PDIA3	1.8062253	0.3451641	0.081237	4.2488537	2.15E-05	0.0028753	TRUE
ANXA2	6.245134	0.1718011	0.0521338	3.2953894	9.83E-04	0.0366269	TRUE
HACD3	1.3302982	0.3683997	0.1006156	3.6614557	2.51E-04	0.0148183	TRUE
RPS17	10.169854	-0.1474088	0.0457124	-3.2246997	0.001261	0.0417138	TRUE
SEZ6L2	1.3528829	0.2519862	0.0755579	3.3350068	8.53E-04	0.0334309	TRUE
TPPP3	0.8209401	0.4733391	0.1305128	3.6267649	2.87E-04	0.0159417	TRUE
RPAIN	2.25447	0.2031108	0.0615939	3.297581	9.75E-04	0.0366269	TRUE
PMP22	4.738986	0.3511181	0.0801134	4.3827629	1.17E-05	0.0021562	TRUE
SCPEP1	0.5974893	0.5184787	0.1141952	4.5402838	5.62E-06	0.0015035	TRUE
MXRA7	1.1214389	0.2798657	0.0832684	3.3610089	7.77E-04	0.0313186	TRUE
TIMP2	1.555492	0.3126524	0.0939072	3.3293776	8.70E-04	0.0334309	TRUE
PMAIP1	1.3452775	0.5443707	0.1216577	4.4746098	7.66E-06	0.0017954	TRUE
NOP56	1.1384061	0.311682	0.0912825	3.414475	6.39E-04	0.0264981	TRUE
CHGB	8.2511448	0.4336243	0.0808784	5.3614343	8.26E-08	6.08E-05	TRUE
SNAP25	2.6102838	0.2297737	0.0652018	3.5240405	4.25E-04	0.0195509	TRUE
PHF20	0.7606434	-0.323262	0.1008685	-3.2047868	0.0013516	0.0433522	TRUE
ATP5D	2.2712189	0.1909267	0.057368	3.3281071	8.74E-04	0.0334309	TRUE
UQCR11	3.1146963	0.1705986	0.0540879	3.1541001	0.0016099	0.0498912	TRUE
MT-ND1	16.64741	0.1653614	0.0460522	3.5907398	3.30E-04	0.0167372	TRUE
MT-CO3	33.082217	0.2462432	0.0489132	5.0342876	4.80E-07	2.35E-04	TRUE

Supplementary Table 2 – List of differentially expressed genes between PD46693 minor and major clone tumour cell population.

Log2FoldChange is calculated as log2(minor clone/major clone). A positive log2FoldChange value indicates a higher expression level in the minor clone population.

gene	baseMean	log2FoldChange	lfcSE	stat	pvalue	padj	sig
HES4	0.8253738	-0.4477104	0.1117724	-4.005553	6.19E-05	0.0040649	TRUE
JUN	4.121573	0.2840566	0.0939382	3.0238658	0.0024957	0.0495569	TRUE
BCL11A	0.6173239	-0.5659022	0.1442805	-3.9222359	8.77E-05	0.0040649	TRUE
GATA2	0.8563014	0.4728011	0.1330183	3.5544059	3.79E-04	0.0131645	TRUE
HMX1	1.1620017	0.3751426	0.1113263	3.3697572	7.52E-04	0.0209152	TRUE
ISL1	1.46513	0.2864101	0.0913407	3.1356239	0.0017149	0.0397283	TRUE
CDC5L	0.797059	0.4361822	0.1017803	4.2855281	1.82E-05	0.002534	TRUE

Supplementary Table 3 – List of transcription factors that are differentially expressed between PD46693 minor and major clone tumour cell population.

Log2FoldChange is calculated as log2(minor clone/major clone). A positive log2FoldChange value indicates a higher expression level in the minor clone population.

Supplementary References

1. Young, M. D. et al. Single-cell transcriptomes from human kidneys reveal the cellular identity of renal tumors. Science 361, 594–599 (2018).
2. Kildisiute, G. et al. Tumor to normal single-cell mRNA comparisons reveal a pan-neuroblastoma cancer cell. Sci. Adv. 7, eabd3311 (2021).
3. Young, M. D. et al. Single cell derived mRNA signals across human kidney tumors. Nat. Commun. 12, 1–19 (2021).
