# Supplementary material for: Precise identification of cancer cells from allelic imbalances in single cell transcriptomes
Source: Commun Biol. 2022 Sep 7;5:884. doi: 10.1038/s42003-022-03808-9 (PMC9452529; doi:10.1038/s42003-022-03808-9)
Supplement: Supplementary file 2 — Description of Additional Supplementary Files [file 42003_2022_3808_MOESM2_ESM.pdf]

Description of Additional Supplementary Files

File name: Supplementary Data 1
Description: WGS-derived copy number profile per individual as identified by Battenberg.

File name: Supplementary Data 2
Description: Numerical values used in generating the figures in the manuscript.
