# Supplementary material for: Precise identification of cancer cells from allelic imbalances in single cell transcriptomes
Source: Commun Biol. 2022 Sep 7;5:884. doi: 10.1038/s42003-022-03808-9 (PMC9452529; doi:10.1038/s42003-022-03808-9)
Supplement: Supplementary file 5 — Reporting Summary [file 42003_2022_3808_MOESM5_ESM.pdf]

nature research

Corresponding author(s): Matthew D. Young, Sam Behjati

Last updated by author(s): Jun 28, 2022

Reporting Summary

Nature Research wishes to improve the reproducibility of the work that we publish. This form provides structure for consistency and transparency in reporting. For further information on Nature Research policies, see our Editorial Policies and the Editorial Policy Checklist.

Statistics

For all statistical analyses, confirm that the following items are present in the figure legend, table legend, main text, or Methods section.

n/a	Confirmed	
☒	☐	The exact sample size (*n*) for each experimental group/condition, given as a discrete number and unit of measurement
☒	☐	A statement on whether measurements were taken from distinct samples or whether the same sample was measured repeatedly
☐	☒	The statistical test(s) used AND whether they are one- or two-sided<br>*Only common tests should be described solely by name; describe more complex techniques in the Methods section.*
☐	☒	A description of all covariates tested
☐	☒	A description of any assumptions or corrections, such as tests of normality and adjustment for multiple comparisons
☒	☐	A full description of the statistical parameters including central tendency (e.g. means) or other basic estimates (e.g. regression coefficient) AND variation (e.g. standard deviation) or associated estimates of uncertainty (e.g. confidence intervals)
☒	☐	For null hypothesis testing, the test statistic (e.g. *F*, *t*, *r*) with confidence intervals, effect sizes, degrees of freedom and *P* value noted<br>*Give P values as exact values whenever suitable.*
☐	☒	For Bayesian analysis, information on the choice of priors and Markov chain Monte Carlo settings
☒	☐	For hierarchical and complex designs, identification of the appropriate level for tests and full reporting of outcomes
☒	☐	Estimates of effect sizes (e.g. Cohen's *d*, Pearson's *r*), indicating how they were calculated

Our web collection on statistics for biologists contains articles on many of the points above.

Software and code

Policy information about availability of computer code

Data collection	No software was required to collect data
Data analysis	CaVEMan (1.14.0; https://github.com/cancerit)<br>ASCAT (4.3.3; https://github.com/cancerit)<br>Battenberg (3.5.2; https://github.com/cancerit)<br>Scrublet (0.2.3; https://github.com/swolock/scrublet)<br>Seurat (4.1.0; https://github.com/satijalab/seurat)<br><br>alleleIntegrator (0.7.3; https://github.com/constantAmateur/alleleIntegrator)<br>CopyKAT (1.0.8; https://github.com/navinlabcode/copykat)<br>inferCNV (1.6.0; https://github.com/broadinstitute/infercnv)<br><br>R (4.0.3)<br><br>Bespoke R scripts used for analysis and visualisation in this study are available online from GitHub (https://github.com/mitrinh1/scGenotyping).

For manuscripts utilizing custom algorithms or software that are central to the research but not yet described in published literature, software must be made available to editors and reviewers. We strongly encourage code deposition in a community repository (e.g. GitHub). See the Nature Research guidelines for submitting code & software for further information.

Data

Policy information about availability of data

All manuscripts must include a data availability statement. This statement should provide the following information, where applicable:

- Accession codes, unique identifiers, or web links for publicly available datasets
- A list of figures that have associated raw data
- A description of any restrictions on data availability

Three previously published single-cell RNAseq dataset was obtained for this study:
1. Renal cell carcinoma: Young, M. D. et al. Single-cell transcriptomes from human kidneys reveal the cellular identity of renal tumors. Science 361, 594–599 (2018)
2. Neuroblastoma: Kildisiute, G. et al. Tumor to normal single-cell mRNA comparisons reveal a pan-neuroblastoma cancer cell. Sci. Adv. 7, eabd3311 (2021).
3. Wilms tumour: Young, M. D. et al. Single cell derived mRNA signals across human kidney tumors. Nat. Commun. 12, 1–19 (2021).

Newly generated single-cell RNAseq datasets (Ewing's sarcoma and AT/RT) has been deposited in the EGA under accession code EGAD00001009005.

Copy number profiles for individual samples derived from whole genome sequencing as called by Battenberg can be found in supplementary tables 4-16, as well as Figure S2,4-5.

There will be no restrictions on data availability.

Field-specific reporting

Please select the one below that is the best fit for your research. If you are not sure, read the appropriate sections before making your selection.

☒ Life sciences ☐ Behavioural & social sciences ☐ Ecological, evolutionary & environmental sciences

For a reference copy of the document with all sections, see nature.com/documents/nr-reporting-summary-flat.pdf

Life sciences study design

All studies must disclose on these points even when the disclosure is negative.

Sample size

No sample size calculation was performed.

The published single-cell RNA-seq datasets used in this study were selected based on:
1. the presence of copy number aberrations in the cancer lineage
2. the presence of cancer-derived and non cancer-derived cells from both tumour and normal biopsies from multiple different patients
3. the wider field interest in which cell types are cancerous in Neuroblastoma

The newly generated single-cell RNA-seq data was determined by the availability of tumour biopsies where genomic copy number aberrations were observed, as well as the cost of the experiment.

This study is not dependent on establishing statistical significant differences among patients, and therefore does not require sample size calculation.

Data exclusions

Low quality cells from all single-cell RNA-seq datasets were excluded from downstream analysis at QC steps. The exact QC filtering criteria are detailed in the "Methods" section.

Additionally, some minor cell types from the original renal cell carcinoma (RCC) and neuroblastoma (NB) single-cell RNA-seq datasets were excluded. This was pre-established, where only relevant interesting cell types were chosen to ensure a focused analysis. Leukocytes and tumour population from both datasets were retained as they are definitively known normal and tumour cells, respectively. For RCC, proximal tubular cells were included as they are probable cell of origin for RCC, but are not cancer-derived as they come from normal tissue biopsies. For NB, endothelial cells are another normal population, whereas it has been heavily debated whether mesenchymal cells are cancerous.

Replication

As the single-cell RNA-seq data was generated using fresh biopsy samples, it was not possible to have technical replicates with limited amount of materials.

Randomization

This study does not involve experimental groups.

Blinding

This study does not involve experimental groups.

Reporting for specific materials, systems and methods

We require information from authors about some types of materials, experimental systems and methods used in many studies. Here, indicate whether each material, system or method listed is relevant to your study. If you are not sure if a list item applies to your research, read the appropriate section before selecting a response.

Materials & experimental systems

n/a	Involved in the study
☒	☐ Antibodies
☒	☐ Eukaryotic cell lines
☒	☐ Palaeontology and archaeology
☒	☐ Animals and other organisms
☐	☒ Human research participants
☒	☐ Clinical data
☒	☐ Dual use research of concern

Methods

n/a	Involved in the study
☒	☐ ChIP-seq
☒	☐ Flow cytometry
☒	☐ MRI-based neuroimaging

Human research participants

Policy information about studies involving human research participants

Population characteristics	The relevant patient covariate to this study was tumour type, which is listed for each individual in Supplementary Table 1.
Recruitment	Human tumour tissues were collected through studies approved by UK NHS research ethics committees. Patients or guardians provided informed written consent for participation in this study as stipulated by the study protocols.
Ethics oversight	This study has the reference NHS National Research Ethics Service reference 16/EE/0394 (paediatric tissues).

Note that full information on the approval of the study protocol must also be provided in the manuscript.
